# The economic impact of substandard and falsified antimalarial medications in Nigeria

**DOI:** 10.1371/journal.pone.0217910

**Published:** 2019-08-15

**Authors:** Sarah M. Beargie, Colleen R. Higgins, Daniel R. Evans, Sarah K. Laing, Daniel Erim, Sachiko Ozawa

**Affiliations:** 1 Division of Practice Advancement and Clinical Education, UNC Eshelman School of Pharmacy, University of North Carolina, Chapel Hill, North Carolina, United States of America; 2 Duke University School of Medicine, Durham, North Carolina, United States of America; 3 Quality Measurement and Health Policy Group, RTI International, Research Triangle Park, North Carolina, United States of America; 4 Department of Maternal and Child Health, UNC Gillings School of Global Public Health, University of North Carolina, Chapel Hill, North Carolina, United States of America; Instituto Rene Rachou, BRAZIL

## Abstract

**Introduction:**

Substandard and falsified medications pose significant risks to global health. Nearly one in five antimalarials circulating in low- and middle-income countries are substandard or falsified. We assessed the health and economic impact of substandard and falsified antimalarials on children under five in Nigeria, where malaria is endemic and poor-quality medications are commonplace.

**Methods:**

We developed a dynamic agent-based SAFARI (Substandard and Falsified Antimalarial Research Impact) model to capture the impact of antimalarial use in Nigeria. The model simulated children with background characteristics, malaria infections, patient care-seeking, disease progression, treatment outcomes, and incurred costs. Using scenario analyses, we simulated the impact of substandard and falsified medicines, antimalarial resistance, as well as possible interventions to improve the quality of treatment, reduce stock-outs, and educate caregivers about antimalarial quality.

**Results:**

We estimated that poor quality antimalarials are responsible for 12,300 deaths and $892 million ($890-$893 million) in costs annually in Nigeria. If antimalarial resistance develops, we simulated that current costs of malaria could increase by $839 million (11% increase, $837-$841 million). The northern regions of Nigeria have a greater burden as compared to the southern regions, with 9,700 deaths and $698 million ($697-$700 million) in total economic losses annually due to substandard and falsified antimalarials. Furthermore, our scenario analyses demonstrated that possible interventions—such as removing stock-outs in all facilities ($1.11 billion), having only ACTs available for treatment ($594 million), and 20% more patients seeking care ($469 million)—can save hundreds of millions in costs annually in Nigeria.

**Conclusions:**

The results highlight the significant health and economic burden of poor quality antimalarials in Nigeria, and the impact of potential interventions to counter them. In order to reduce the burden of malaria and prevent antimalarials from developing resistance, policymakers and donors must understand the problem and implement interventions to reduce the impact of ineffective and harmful antimalarials.

## Introduction

Malaria is endemic in Nigeria where the entire country’s 191 million residents are at risk [1, 2]. *Plasmodium falciparum* causes an estimated 99.7% of deaths due to malaria with a disproportionate number of deaths in children under five [1, 3]. In 2017, Nigeria had an estimated 53.7 million cases of malaria across all ages, which accounted for 25% of all clinical episodes of malaria worldwide [1]. Furthermore, 19% of the global estimate of malaria deaths (81,600 deaths in 2017) occurred in Nigeria, making Nigeria the single most malaria-burdened country in the world [1, 4].

In addition to the high burden of malarial disease, the quality of antimalarial treatments available in Nigeria further impact its health and economy. Antimalarials are one of the most commonly found medications to be substandard or falsified in low- and middle-income countries (LMICs) including in Nigeria [5]. According to a recent meta-analysis, 19.1% of all antimalarials tested in LMICs were substandard or falsified [5]. The World Health Organization (WHO) defines substandard medicines as authorized medical products that fail to meet either quality standards, specifications, or both [6]. Falsified medicines are medical products that deliberately or fraudulently misrepresent their identity, composition or source [6]. As a result of poor manufacturing, inadequate supply chain management and storage conditions, or sales beyond expiration, poor quality medications can contain sub-therapeutic concentrations of stated ingredients, improper ingredients or no active pharmaceutical ingredients [4, 7].

Poor quality antimalarial medications place significant health, social, and economic burden on individuals and communities. Treatment failures prolong malaria illness and result in avertable costs. Substandard and falsified antimalarials can extend the length of hospitalization, contribute to antimalarial resistance, and even lead to death [6, 8, 9]. In addition to health effects, poor quality antimalarials impose avoidable economic costs to patients, their families and the health system, through costs of medical care and productivity losses [10, 11]. Substandard and falsified antimalarials can also increase health inequities [12].

The impact of poor quality antimalarials on under-five deaths has been estimated at the regional level in sub-Saharan Africa [13]. We have presented detailed country-level estimates for Uganda [14] and the Democratic Republic of the Congo [15], but no such analyses have focused on Nigeria or countries in west Africa to date. This project estimates the health and economic impact of substandard and falsified antimalarials in Nigeria overall, and in the northern and southern regions. It also assesses the effects of potential interventions to inform policy decisions to improve the quality of treatment, reduce stock-outs, and educate caregivers about antimalarial quality.

## Materials and methods

The SAFARI (Substandard and Falsified Antimalarial Research Impact) model is an agent-based model used to estimate the health and economic impact of substandard and falsified antimalarials on children under five [14, 15]. The methods for the development of the SAFARI model are described in detail in other publications [14, 15], with adaptations specific to Nigeria outlined here. The SAFARI model was built in Python to simulate population characteristics, malaria infection, patient care-seeking, disease progression, treatment outcomes, and associated costs of malaria. Agent-based models capture greater heterogeneity in the flow and actions of agents by incorporating individual characteristics, as opposed to a Markov model or a decision tree that assume population groups are homogenous. Heterogeneity is incorporated into the model through characteristics ascribed to each of the 25,000 simulated child agents, including demographic characteristics, individual incidence, and care-seeking probabilities. These characteristics drive the actions of agents and allow for a more granular analysis of the results. Four demographic characteristics—geographic region, rural/urban, wealth quintile, and level of maternal education—were applied to each child in the model, according to the distributions from the most recent (2015) Nigeria Malaria Indicator Survey (MIS) [16]. We also adjusted for regional variations in malaria transmission in Nigeria, with higher transmission in the northern region as compared to the southern region. This was incorporated through each agent’s individual probability of becoming ill with malaria, reflecting the prevalence of malaria by region.

The model flow diagram found in S1 Fig depicts the flow of the SAFARI model for Nigeria. The model simulated a one-year time horizon in five-day increments based on the reported average duration of an uncomplicated malaria case, accounting for time to seek care and average duration of symptoms [17]. All agents (simulated children under five) moved through the disease and care-seeking simulations based on their individual background characteristics. Agents became infected and symptomatic based on estimates of under-five malaria incidence and cases in Nigeria [1, 18, 19].

We simulated malaria treatment at one of six locations: public facilities, private facilities, pharmacies/chemists, drug stores/drug hawkers/general retailers, community health workers (CHWs), or self-treatment. We also observed the progression of the disease among individuals not seeking care. Antimalarial treatment available in each location was based on the market share of three treatment options: artemisinin-based combination therapies (ACTs), chloroquine, or other treatments (such as sulfadoxine-pyrimethamine (SP), amodiaquine, quinine and others) [16]. Each care location could run out of stock of antimalarials based on national stock data from ACTwatch [20], where non-severe cases remained symptomatic through the next period. Child agents could progress to severe malaria and then face the probability of dying while receiving treatment at hospitals, or dying without receiving treatment in the community [21]. To account for adverse outcomes caused by substandard or falsified antimalarials, it was assumed that patients who received poor quality antimalarials faced a 50% increase in the probability of developing severe malaria, reflecting the impact of reduced efficacy of antimalarials with lower amounts of active pharmaceutical ingredients (API) [6].

A literature review was conducted in November 2017 across five electronic databases [PubMed, EconLit, Global Health, Embase, and SCOPUS] to identify model inputs specific to Nigeria. Grey literature was also searched to identify inputs from sources such as ACTwatch, Global Burden of Disease, Malaria Atlas Project (MAP), Nigeria MIS, World Malaria Report, World Development Indicators, and the Worldwide Antimalarial Resistance Network (WWARN) [2, 18, 20, 22–30]. Data inputs were chosen based on their quality, relevance, and generalizability for the most recent year. The main demographic, epidemiological, and cost inputs are outlined in Table 1, with additional coefficients included in S1 Appendix. In order to account for natural variations in epidemiological and cost inputs, key data were ranged and simulated to vary probabilistically. Epidemiological data were varied based on beta distributions and cost data were ranged using gamma distributions. Costs in Nigerian Naira were converted to 2017 USD using local inflation rates and 2017 exchange rates from the Central Bank of Nigeria [31].

**Table 1:** Key data inputs for the SAFARI model in Nigeria.

	Model Inputs	Estimate	Range	Source
**Demographic & Epidemiological Data**	**<5 Population at Risk**	32,379,000		[32]
	**Malaria Incidence**	0.8096		[18]
	**Inpatient Severe Malaria Cases (per 100,000 people per year)**	44.8		Calibrated with: [21]
	**Probability for Treatment Failure to Progress to Severe**	0.020	(0.005–0.05)	[26]
	**Inpatient Severe Case Fatality Rate**	0.08		[21]
	**Severe Case Fatality Rate in the Community**	0.15		[21]
	**Case Fatality Rate Without Receiving Treatment**	0.6	(0.45–0.8)	[22]
	**Probability of Neurological Sequelae for Inpatient Severe Case**	0.0313	(0.028–0.035)	Calibrated with: [27]
**Healthcare Seeking Behavior**	**Care-Seeking Behavior (%)**			[16]
	Public Facilities	19.9%		
	Private Facilities	7.0%		
	Pharmacies/Chemists	39.1%		
	Drug Stores/Drug Hawkers/General Retailers	0.8%		
	CHWs	0.9%		
	Self/Neighbors	20.2%		
	No Treatment	12.3%		
**Medication Stock by Facility**	**Public Facilities**			[16]
	% Stock ACTs	48.5%		
	% Stock Chloroquine	25.1%		
	% Stock Other Treatments	25.4%		
	**Private Facilities**			
	% Stock ACTs	48.1%		
	% Stock Chloroquine	22.8%		
	% Stock Other Treatments	29.1%		
	**Pharmacies/Chemists**			
	% Stock ACTs	36.7%		
	% Stock Chloroquine	30.8%		
	% Stock Other Treatments	32.6%		
	**Drug Stores/Drug Hawkers/General Retailers**			
	% Stock ACTs	53.8%		
	% Stock Chloroquine	7.7%		
	% Stock Other Treatments	38.5%		
	**CHWs**			
	% Stock ACTs	54.5%		
	% Stock Chloroquine	0.0%		
	% Stock Other Treatments	45.5%		
	**Self/Neighbors**			
	% Stock ACTs	29.1%		
	% Stock Chloroquine	32.1%		
	% Stock Other Treatments	38.8%		
**Probability of stock-out**	**Proportion of facilities without stock of ACTs**			[20]
	Public Facilities	12.7%		
	Private Facilities	25.5%		
	Pharmacies/Chemists	0.1%		
	Drug Store/Drug Hawkers/General Retailers	11.6%		
	CHWs	0%		Assumption
	Self/Neighbors	0%		
**Medication Effectiveness**	**ACT Effectiveness**	0.9643	(0.9599–0.9687)	Estimated based on: [33], [34], [35]
	**Chloroquine Effectiveness**	0.5444	(0.4246–0.7194)	Estimated based on: [36], [37]
	**Other Treatment Effectiveness**	0.7266	(0.6731–0.7801)	Estimated based on: [36–43]
	**No Treatment Effectiveness**	0		Assumption
**Medication Costs by Facility**	**Public Facilities**			
	Average Cost of ACTs	$0.00		[20]
	Average Cost of Chloroquine	$0.00		
	Average Cost of Other Treatments	$0.00		
	**Private Facilities**			
	Average Cost of ACTs	$2.10	($1.53 –$2.67)	[20]
	Average Cost of Chloroquine	$0.41	($0 –$0.91)	
	Average Cost of Other Treatments	$1.40	($0.61 –$2.19)	
	**Pharmacies/Chemists**			
	Average Cost of ACTs	$3.25	($2.69 –$3.81)	[20]
	Average Cost of Chloroquine	$0.51	($0.01 –$1.01)	
	Average Cost of Other Treatments	$1.47	($0.71 –$2.23)	
	**Drug Stores/Drug Hawkers/General Retailers**			
	Average Cost of ACTs	$2.08	($1.66 –$2.50)	[20]
	Average Cost of Chloroquine	$0.25	($0 –$0.75)	
	Average Cost of Other Treatments	$1.47	($0.71 –$2.23)	
	**CHWs**			
	Average Cost of ACTs	$0.00		[20]
	Average Cost of Chloroquine	$0.00		
	Average Cost of Other Treatments	$0.00		
	**Self/Neighbors**			
	Average Cost of ACTs	$0.00		Assumption
	Average Cost of Chloroquine	$0.00		
	Average Cost of Other Treatments	$0.00		
**Non-Medication Costs**	**Median Cost per Hospitalization**	$10.24	($2.04 –$18.44)	[29]
	**Median Testing Costs**	$1.11	($0.87–1.35)	Estimated based on: [20]
	**Average Transportation Costs**	$1.09		[28–30]
	**Productivity Losses Per Sick Day**	$6.30		Estimated based on: [23]
	**Productivity Losses per Death**	$52,554.65		Estimated based on: [23]
**Proportions of SF Medications**	**ACTs**			
	Not SF (API > 85%)	0.882		Estimated based on: [4, 44–48]
	Category 1: API = 75–85%	0.064		Estimated based on: [4, 20, 44–50]
	Category 2: API = 50–75%	0.027		
	Category 3: API < 50%	0.027		
	**Chloroquine**			
	Not SF (API > 85%)	-.494		Estimated based on: [47, 51–53]
	Category 1: API = 75–85%	0.273		Estimated based on: [4, 20, 47–50]
	Category 2: API = 50–75%	0.118		Estimated based on: [4, 20, 47–50]
	Category 3: API < 50%	0.116		
	**Other Treatments**			
	Not SF (API > 85%)	0.479		Estimated based on: [47, 48, 51–53]
	Category 1: API = 75–85%	0.281		Estimated based on: [4, 20, 47–50]
	Category 2: API = 50–75%	0.121		Estimated based on: [4, 20, 47–49, 51, 53]
	Category 3: API < 50%	0.119		Estimated based on: [4, 20, 47–53]

ACTs—Artemisinin-based combination therapy; API—Active pharmaceutical ingredient; CHWs—community health workers; SF—substandard and falsified

The treatment outcome for each agent in the model was determined based on treatment adherence rates, treatment efficacy by medication, and the API concentration of the specific treatment the agent-child received [33–44, 46, 49–54]. Treatment efficacy and prevalence of substandard and falsified medicines for each antimalarial treatment were estimated with data extracted from the WWARN database and prevalence studies specific to Nigeria [33–43, 47, 48, 51]. Each modeled antimalarial medication was assigned an API percentage category (>85%, 75–85%, 50–75% and <50%) and given a corresponding treatment efficacy where lower API levels reduced treatment effectiveness. Each agent in the model was assigned a probability of treatment adherence, which also affected treatment outcomes [55].

The primary model outputs are estimates of the health impact, direct costs, and productivity losses attributable to substandard and falsified antimalarials taken among children under five in Nigeria. The health impact is presented as the number of uncomplicated and severe cases, neurological sequelae, and deaths due to malaria. Economic outputs assessed direct costs for consultation, medications, transportation, hospitalization and testing, as well as productivity losses. Consultation costs included the cost to the patient and facility of supplemental medicines or food, additional increased costs of private facility care, and the cost of health care services excluding medication and testing. Productivity losses included lost caretaker time caring for sick children and long-term productivity losses for patients over a lifetime due to malaria-induced disability or premature death. Direct costs were further separated into amounts paid by patients and caretakers out-of-pocket versus those incurred by health facilities.

We compared the baseline estimate to a scenario with no substandard and falsified antimalarials (i.e. assuming all medicines have an API > 85%) to assess the added expenses of poor quality medications. In addition, we present a hypothetical scenario where *Plasmodium falciparum* has developed resistance to artemisinin-based antimalarials, where treatment efficacies for ACTs were lowered to be the same as those for other treatments. We present the health and economic outputs separately for northern and southern regions of Nigeria. In addition to the main simulations, seven other scenarios of potential interventions were examined. These scenarios were chosen to represent various supply chain, antimalarial treatment policies and caregiver education interventions. The scenarios included: having no medication stock-outs (1) across all sectors, (2) in public facilities, or (3) in private facilities; (4) replacing chloroquine and other treatments with ACTs such that only ACTs are available for treatment; (5) replacing all substandard or falsified ACTs with good quality ACTs; (6) encouraging 20% more patients to seek care for malaria treatment; and (7) encouraging perfect adherence to antimalarial medications.

## Results

Annually, we simulated approximately 24 million cases of malaria in children under five in Nigeria. Of cases that progressed to severe, we estimated 147,000 hospitalizations, 8,200 cases of neurological sequelae, and 78,000 deaths per year. The total economic impact of malaria in Nigeria was estimated at $7.76 billion (7.73–7.80 billion) with $7.36 billion (95% of total economic impact, 7.33–7.40 billion) in productivity losses, including $4.1 billion in lifetime productivity losses and $3.08 billion in short-term productivity losses. Direct costs of seeking medical treatment for malaria were approximately $401 million (5% of total economic impact, 400.4–401.4 million), which included $316 million for consultation costs, $59.3 million for medication costs, $9.5 million for transportation costs, $8.97 million for hospitalization costs, and $7.5 million for testing costs. Up to 33% of the direct costs of malaria treatment ($134 million) were paid out-of-pocket, whereas the health facility incurred the remainder of the costs ($267 million). The health and economic burden of malaria in Nigeria is summarized in Table 2.

**Table 2:** Estimated burden of malaria, the health and economic impact of substandard and falsified antimalarials, and effect of antimicrobial resistance of ACTs in Nigeria.

		Burden of Malaria		No Substandard or Falsified Antimalarials			Antimicrobial Resistance		
		Baseline	95% CI	Potential Savings	Percent Difference	p-value**	Additional Costs	Percent Difference	p-value**
**Health Impact**	Average Number of Cases	24,000,000	(23,995,800–24,002,700)						
	Average Number Hospitalized	147,000	(146,900–147,700)	-33,300	-23%	<0.001	+19,200	+13%	<0.001
	Average Number with NS	8,200	(8,100–8,200)	-500	-6%	<0.001	+800	+10%	<0.001
	Average Number of Deaths	78,000	(77,800–78,300)	-12,300	-16%	<0.001	+7,700	+10%	<0.001
**Economic Impact**	Total Economic Impact	$7,760,000,000	(7,729,178,500–7,800,795,900)	-$892,000,000	-11%	<0.001	+$839,000,000	+11%	<0.001
	Direct Costs	$401,000,000	(400,398,700–401,399,000)	-$29,800,000	-7%	<0.001	+$44,600,000	+11%	<0.001
	Facility Costs	$267,000,000	(266,997,100–267,799,200)	-$20,000,000	-7%	<0.001	+$29,900,000	+11%	<0.001
	Out-of-Pocket Costs	$134,000,000	(133,206,400–133,795,100)	-$9,800,000	-7%	<0.001	+$14,800,000	+11%	<0.001
	All Productivity Losses	$7,360,000,000	(7,328,289,900–7,399,886,800)	-$862,000,000	-12%	<0.001	$794,000,000	+11%	<0.001
	Short-Term*	$3,080,000,000	(3,042,502,100–3,109,500,200)	-$203,000,000	-1%	<0.001	+$369,000,000	+12%	<0.001
	Lifetime	$4,100,000,000	(4,086,615,200–4,113,241,200)	-$648,000,000	-16%	<0.001	+$405,000,000	+10%	<0.001

ACT—Artemisinin-based combination therapies; CI—Confidence interval; NS—Neurological sequelae

* Short-Term productivity losses included caregiver time during care seeking and hospital stay, and opportunity costs incurred by the community-health worker program. Lifetime productivity losses included losses due to premature death and disability.

** Unpaired t-tests estimated the statistical significance of outputs (p<0.05) compared to baseline.

Substandard and falsified antimalarials contributed significantly to the malaria burden in Nigeria. Our literature review found that 11.8% of ACTs were substandard or falsified, and 14.1% of all medicines overall in Nigeria were of poor quality. Replacing poor quality antimalarials with good quality ones resulted in 33,300 fewer hospitalizations and 12,300 fewer deaths annually in the country. The annual economic impact of substandard and falsified antimalarials in Nigeria was estimated at $892 million ($890-$893 million), comprising about 11% of the total economic burden of malaria. This included $648 million ($647.9–649.1 million) in lifetime productivity losses and $203 million ($202-$205 million) in short-term productivity losses each year as a result of poor quality antimalarials. Substandard and falsified antimalarials accounted for $29.85 million ($29.82-$29.87 million) in direct costs annually, including $9.8 million ($9.78-$9.81 million) as out-of-pocket costs to patients who sought care.

If artemisinin resistance were to emerge reducing the effectiveness of ACTs to the level of other treatments, we estimated that Nigeria could face 19,200 more hospitalizations and 7,700 additional deaths among patients under five seeking treatment each year. In our simulation, antimalarial resistance increased costs to Nigeria by $839 million ($837-$841 million) annually, an additional 11% increase in the economic burden of malaria. This included increases in lifetime productivity losses by $405 million ($404.8-$406 million), short-term productivity losses by $369 million ($368-$371 million), and direct costs by $44.65 million ($44.63-$44.67 million). We estimated that antimalarial resistance could add $29.89 million ($29.87-$29.91 million) to health facility costs and $14.76 million ($14.75-$14.78 million) in out-of-pocket costs to patients who seek treatment every year, resulting in an 11% increase in direct costs.

Northern and southern regional results are presented in Table 3. The economic burden of malaria was found to be much greater in the northern region of Nigeria at $6.09 billion ($6.06-$6.11 billion) as compared to $1.68 billion ($1.67-$1.69 billion) in the southern region. Much of the difference between regions can be explained by malaria transmission rates, with populations in the northern region at greater risk of malaria. Our model simulated 18.9 million malaria cases (79% of all cases) in Nigeria’s northern region, where 65% of all children under five live. In the southern region, we estimated 5.09 million cases (21% of all cases) of malaria. We estimated that 261,000 cases per year in the northern region advance to severe malaria leading to 61,000 deaths, compared to 70,500 severe cases and 17,100 deaths in the southern region.

**Table 3:** The health and economic impact of substandard and falsified antimalarials in Nigeria: Northern vs. southern regions.

**Northern Region**									
		**Burden of Malaria**		**No Substandard or Falsified Antimalarials**			**Antimicrobial Resistance**		
		**Baseline**	**95% CI**	**Potential Impact**	**Percent Difference**	**p-value**	**Additional Costs**	**Percent Difference**	**p-value**
**Health Impact**	Average Number of Cases	18,900,000	(18,905,600–18,912,500)						
	Average Number Hospitalized	116,000	(115,900–116,600)	-26,300	-23%	<0.001	+14,900	+13%	<0.001
	Average Number of Deaths	61,000	(60,700–61,200)	-9,700	-16%	<0.001	+6,000	+10%	<0.001
**Economic Impact**	Total Economic Impact	$6,090,000,000	($6,057,711,900 –$6,114,788,000)	-$698,000,000	-11%	<0.001	+$653,000,000	+11%	<0.001
	Facility Costs	$209,000,000	($208,768,500 –$209,401,200)	-$15,600,000	-7%	<0.001	+$23,100,000	+11%	<0.001
	All Productivity Losses	$5,770,000,000	($5,743,514,900 –$5,800,573,400)	-$675,000,000	-12%	<0.001	+$619,000,000	+11%	<0.001
	Out-of-Pocket Costs	$105,000,000	($104,899,200 –$105,352,800)	-$7,660,000	-7%	<0.001	+$11,500,000	+11%	<0.001
**Southern Region**									
		**Burden of Malaria**		**No Substandard or Falsified Antimalarials**			**Antimicrobial Resistance**		
		**Baseline**	**95% CI**	**Cost Savings**	**Percent Difference**	**p-value**	**Additional Costs**	**Percent Difference**	**p-value**
**Health Impact**	Average Number of Cases	5,090,000	(5,088,000–5,092,400)						
	Average Number Hospitalized	31,000	(30,900–31,200)	-6,900	-22%	<0.001	+4,300	+14%	<0.001
	Average Number of Deaths	17,100	(17,000–17,100)	-2,700	-16%	<0.001	+1,700	+10%	<0.001
**Economic Impact**	Total Economic Impact	$1,680,000,000	($1,669,991,000 –$1,687,483,600)	-$193,000,000	-12%	<0.001	+$185,000,000	+11%	<0.001
	Facility Costs	$58,300,000	($58,218,600 –$58,408,100)	-$4,480,000	-8%	<0.001	+$6,740,000	+12%	<0.001
	All Productivity Losses	$1,590,000,000	($1,583,302,900 –$1,600,785,600)	-$187,000,000	-12%	<0.001	+$175,000,000	+11%	<0.001
	Out-of-Pocket Costs	$28,400,000	($28,315,100 –$28,444,300)	-$2,140,000	-8%	<0.001	+$3,250,000	+11%	<0.001

CI—Confidence interval

The burden of substandard and falsified medicines was especially large in northern Nigeria, where their removal in the northern region would save $698 million ($697-$700 million) annually, in contrast to $193 million ($193.1-$193.8 million) in annual savings in the southern region. On the other hand, if antimalarial resistance to ACTs were to emerge, we estimated that total costs of malaria would increase by $653 million ($652-$655 million) in the northern region and $185 million ($185.0-$185.8 million) in the southern region.

**Fig 1:**
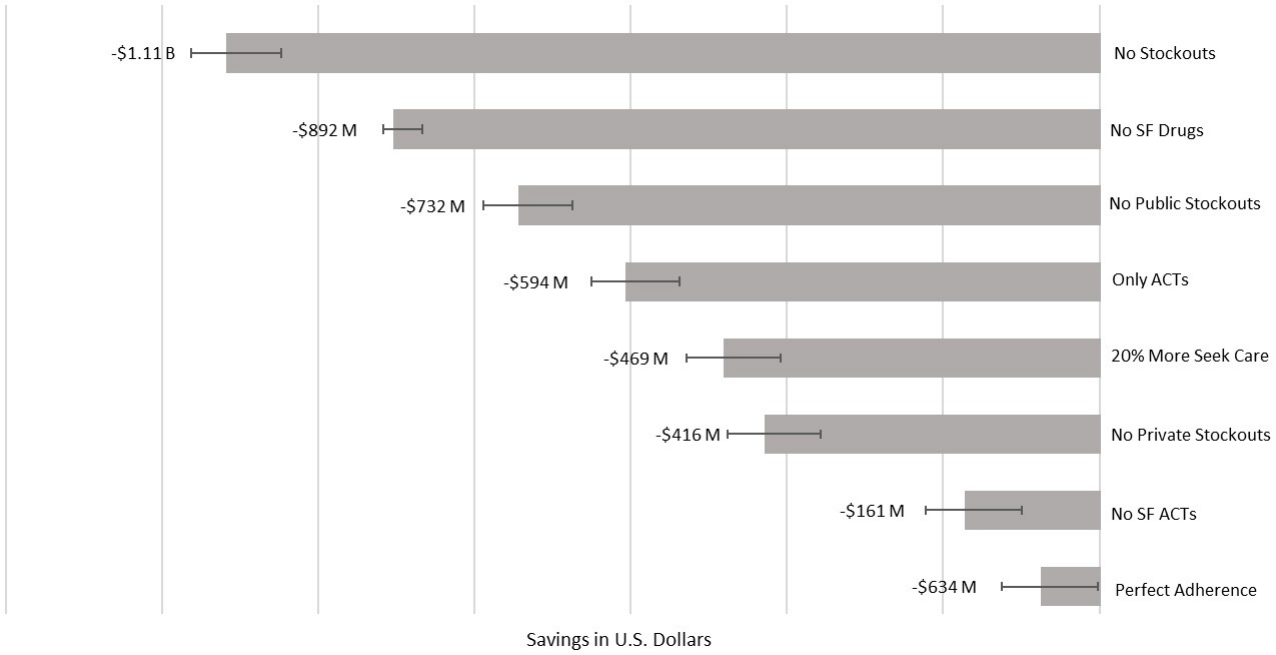
Total economic impact of intervention scenarios.

Fig 1 presents the impact that various interventions could have vis-à-vis improving the quality of antimalarial medications in Nigeria. Eliminating all stock-outs provided the largest cost-savings simulated at $1.11 billion ($1.10-$1.11 billion) annually. Removing all substandard and falsified antimalarials offered the second largest estimated annual savings of $892 million ($890-$893 million). Due to frequent utilization of other treatments, improving only the quality of ACTs but not those of other treatments saved only $161 million ($157-$164 million) in annual costs. When ACTs were the only treatment option available for malaria, replacing chloroquine and other treatments, we estimated $594 million ($591-$698 million) in annual savings. Increasing the number of individuals who seek care for malaria by 20% was estimated to result in $469 million ($465-$473 million) in cost savings. Perfect medication adherence to antimalarials demonstrated a smaller impact ($63 million; $59.6-$67.2 million).

## Discussion

The results demonstrate the threat posed by substandard and falsified antimalarials and the importance of improving access to good quality malaria treatment in Nigeria. Substandard and falsified antimalarials were estimated to be responsible for $892 million ($890–893 million) in costs annually in Nigeria, and was attributable for 6%-23% of the health and economic burden of malaria in the country. If artemisinin resistance were to develop to reduce the effectiveness of ACTs, we simulated that current economic costs could increase by 11% annually ($839 million), including growth in direct costs by 11% ($44.6 million). Improving the quality of antimalarials would make a significant impact in reducing the burden of malaria in Nigeria.

We observed that substandard and falsified antimalarials affect many more children in the northern region (9,700 deaths and $698 million in costs) as compared to the southern region (2,700 deaths and $193 million in costs). This is in line with known regional disparities where northern Nigeria has fewer healthcare providers, weaker infrastructure, more porous supply chains and a larger malaria disease burden. Although the northern region comprises a larger area and population, the southern region has greater availability of financial resources and lower poverty rates, leading to disparities in access to malaria testing, medications, and education about proper treatments [56, 57]. Humanitarian crisis such as the Boko Haram insurgency in the northern region contribute to the increased burden of malaria due to poor disease control, disruptions in the health system, and minimal access to care [32]. Our results suggest that poor quality antimalarials further exacerbate inequities, which was also observed in Uganda [12]. Greater efforts are needed especially in the northern region to protect vulnerable populations and reduce health and economic inequities.

Our results are comparable to previously reported estimates of the malaria disease burden. For example, our model estimated a total of 24 million malaria cases in Nigerian children under five, which is comparable to the approximately 25.8 million under-five malaria cases, based on the WHO estimate that 45% of malaria cases in Nigeria (53.7 million) occur in children under five [6, 32]. Our model estimated a total of 78,000 deaths, which is in line with UNICEF projections that 9.8% of all under-five deaths in Nigeria in 2017 were due to malaria, estimated at 69,990 (50,509–96,460) deaths [58]. Furthermore, our model estimate of the annual burden of malaria in Nigeria at $7.76 billion 2017 USD is comparable to the annual cost of malaria estimated in Nigeria at 2.2 billion in 2011 Naira ($12 billion 2017 USD, 65%) as malaria cases and deaths have comparably lessened over time (2017 estimates were 89% of cases and 60% of deaths in 2011) [59].

Furthermore, the SAFARI model estimates of the impact of substandard and falsified antimalarials on children under five for Nigeria are also in line with previous studies. The WHO decision tree model estimated that substandard and falsified antimalarials are responsible for approximately 2.1%– 4.9% of total malarial deaths in sub-Saharan Africa [6]. A study of country-specific burden of under-five malaria deaths in sub-Saharan Africa estimated a yearly median of 74,188 (interquartile range 54,931–96,132) deaths due to poor quality antimalarials in Nigeria (42% of under-five malaria deaths in Nigeria in 2010) [13]. Our estimate of an annual 12,300 deaths (16% of all under five malaria deaths) due to substandard and falsified antimalarials falls between these estimates, but differences in methodology, scope, and data years makes direct comparisons difficult. The SAFARI model results from Uganda, which has a lower overall burden than Nigeria, found that 8% of total economic impact of malaria is attributable to poor quality antimalarials, compared with the estimate in Nigeria of 12% [14].

Despite efforts by the Nigerian National Agency for Food and Drug Administration and Control (NAFDAC) to manage the supply chain and regulate medicine quality, substandard and falsified medicines continue to proliferate in the Nigerian market. A disorganized network of sellers and weak regulation make the pharmaceutical system in Nigeria particularly vulnerable to unethical and corrupt practices, such as extortion of bribes and diversion of donated medications [60, 61]. Small numbers of pharmaceutical manufacturers in Nigeria are insufficient to meet the local demand, requiring medications to be imported from other countries. The majority of imported medications originate from countries such as China and India, where substandard and falsified medications have been identified [62, 63]. To reduce the total burden of substandard and falsified antimalarials, policymakers should strengthen regulatory capacity to license manufacturers, ensure good manufacturing practices and perform quality control of antimalarials. This could protect malaria medication from threats of falsification, poor manufacturing, expiration, and degradation. The hot and humid conditions in which medications are transported, stored, and sold in these locations often facilitate the degradation of medicines, which can result in substandard effectiveness [64]. In addition, medications frequently expire due to weak distribution systems, making them ineffective. Improving pharmaceutical governance, supply chain management and antimalarial surveillance are essential to close doors to substandard and falsified antimalarials from permeating the supply chain. NAFDAC and the Federal Ministry of Health must coordinate to play a larger role in ensuring quality of medicines by securing supply chains, regulation and inspection, while improving access to high quality medicines.

Increasing access and utilization of ACTs would have a significant health and economic impact in Nigeria. ACTs are recommended as the first-line treatment for malaria by the WHO [32], and Nigeria adopted this recommendation in 2005 [4, 65]. The use of ACTs for malaria treatment in Nigeria increased from 2% in 2008 to 18% in 2013 [2]. Based on the baseline data in our model, we observed that ACTs were used only 36.6% of the time across all care sectors, suggesting that the use of ACTs is far below the national target of 80% by 2010 as specified in the National Malaria Strategic Plan [2, 16]. If ACTs replaced chloroquine, SP and other antimalarial treatments, we simulated that there will be $594 million in total savings and 7,900 fewer deaths among children under five.

The Nigerian supply chain of antimalarials result in frequent stock-outs in public facilities (12.7%) and private facilities (25.5%) [20]. Malaria medicine stock-outs are common due to unmet funding needs, lack of proper training of workers in medicine procurement, and inadequate storage, transportation and distribution practices [64]. Frequent stock-outs push many patients to seek treatment in informal sectors, often receiving antimalarials from drug hawkers, unregistered pharmacies, or open drug markets [61]. Chemists and drug hawkers are often not trained in pharmacy and cannot ensure the legitimacy or safety of medications they sell. Therefore, efforts to empower pharmacists at public and private facilities to better manage antimalarials and reduce stock-outs can prevent patients from purchasing medicines from unregulated markets. Reducing stock-outs of antimalarial medications would not only improve access to malaria treatment, but could also reduce the overall costs of malaria for patients and the government. We simulated that removing stock-outs of antimalarials at public and private facilities resulted in annual savings of $732 million and $416 million in Nigeria, respectively.

Our analyses face a number of key limitations. First, some data inputs were not available by region, which made it difficult to capture the large heterogeneity within Nigeria [66, 67]. Extensive literature searches were conducted and data analyses were carried out to utilize the best and most recent data available for model parameters. To account for some heterogeneity, demographic characteristics were assigned to each agent as well as individual incidence and care-seeking rates based on the analysis of the Nigeria MIS data so that results could be examined separately for northern and southern regions. Data were also not available on the prevalence of substandard and falsified antimalarials by treatment location, where we had to apply the same rates across facility types within the country. In scenario analyses, interventions were examined separately in order to understand their discrete effects. As most of these interventions address a similar population—patients who seek care and utilize antimalarials—the effect of combining interventions would likely be less than additive. In addition, while our scenario analyses examined the impact of various interventions, we did not have data to model the costs of implementing each scenario. Further data should be gathered to inform implementation costs for regulation, quality control, and education to reduce the impact of substandard and falsified medicines. Despite these limitations, we believe this analysis presents important estimates of the health and economic impact of substandard and falsified antimalarials in Nigeria to raise awareness of the problem.

Our results inform the Federal Ministry of Health, NAFDAC, the malaria community and policy makers of the significant burden of substandard and falsified antimalarials in Nigeria. We demonstrate not only the current health and economic burden, but also the benefits that potential interventions could have in reducing this burden. These results should be used to ensure that investments are made to not only guarantee medication safety, but also increase access to high-quality antimalarial treatment. Reducing substandard and falsified antimalarials in Nigeria would decrease the overall malaria burden and also safeguard existing malaria treatments to remain viable from the threat of antimicrobial resistance. The federal government, in collaboration with implementation agencies, international organizations, pharmaceutical companies and healthcare workers should set up efforts to alleviate barriers of access to high-quality ACTs, and strengthen antimalarial supply chains. Improving antimalarial quality is essential to ensure that people can place their trust in medicines and their healthcare system, thereby reducing avertable illnesses, deaths, and costs.

## Supporting information

S1 Appendix Complete input table for the SAFARI model in Nigeria. (DOCX)

S1 Fig Flow diagram for the Nigeria SAFARI model depicting 5 days in a one-year care-seeking cycle. (TIF)
