# Supplementary material for: The economic impact of substandard and falsified antimalarial medications in Nigeria
Source: PLoS One. 2019 Aug 15;14(8):e0217910. doi: 10.1371/journal.pone.0217910 (PMC6695148; doi:10.1371/journal.pone.0217910)
Supplement: S1 Appendix — (DOCX) [file pone.0217910.s001.docx]

**The economic impact of substandard and falsified antimalarial medications in Nigeria**

**Supporting Information File**

**Appendix.** Complete Input Data for the SAFARI Model in Nigeria

| Input | National Value | Range | Source |
| --- | --- | --- | --- |
| *Demographic and Epidemiological Data* | | | |
| Population (<5) in Model | 25,000 |  |  |
| Malaria Incidence (0-59 months old) | 0.80955805 |  | Malaria Atlas Project 2018[1] |
| Case-fatality rate in the community | 0.15 |  | Camponovo 2017[2] |
| Inpatient severe case-fatality rate | 0.08 |  | Camponovo 2017[2] |
| Inpatient Severe Malaria Cases (per 100,000 people per year) | 44.8 |  | Estimated based on: Camponovo 2017[2] |
| Case-fatality Rate (Care Not Sought) | 0.6 | (0.45 – 0.8) | Lubell 2011[3] |
| Neurological Sequelae Rate for patients who received treatment | 0.0313 | (0.028 – 0.035) | Dondorp 2010[4] |
| Neurological Sequelae Rate for patients who did not receive treatment | 0.1936 | (0.145 – 0.258) | Assumption based on: Dondorp 2010[4] |
| Treatment Failure Progression to Severe | 0.020 | (0.005–0.05) | Lubell 2014[5] |
| Probability of Testing |  |  |  |
| Public Facilities | 0.375 |  | Nigeria MIS 2015[6] |
| Private Facilities | 0.365 |  |  |
| Pharmacies/Chemists | 0.066 |  |  |
| Drugstores/Drug Hawkers/General Retailers | 0.081 |  |  |
| CHWs | 0.075 |  |  |
| ACT Cure Rate | 0.9643 | (0.9599 – 0.9687) | Estimated based on: Falade 2005, 4ABC 2011, Falade 2014[7-9] |
| Chloroquine Cure Rate | 0.5444 | (0.4246 – 0.7194) | Estimated based on: Grandesso 2006, Nahum 2007[10, 11] |
| Other Treatment Cure Rate | 0.7266 | (0.6731 – 0.7801) | Estimated based on: Adjuik 2002, Ramharter 2005, Zongo 2005, Grandesso 2006, Nahum 2007, Faucher 2009, Verret 2011, Yeka 2013 [10-17] |
| No Treatment Cure Rate | 0 |  | Assumption |
| Resistance Cure Rate | 0.7266 |  | Assumption |
| Hospital Length of Stay | 4 | 2 – 5 | Assumption |
| Caregiver Length of Care | 5 | 3 – 5 | Assumption |
| Length of Illness (days) | 5 |  |  |
| *MIS Demographic Proportions* |  |  |  |
| Proportion of Children in Region 1 | 0.185 |  | Nigeria MIS 2015[6] |
| Proportion of Children in Region 2 | 0.143 |  |  |
| Proportion of Children in Region 3 | 0.326 |  |  |
| Proportion of Children in Region 4 | 0.088 |  |  |
| Proportion of Children in Region 5 | 0.110 |  |  |
| Proportion of Children in Region 6 | 0.149 |  |  |
| Proportion of Urban Children in Region 1 | 0.201 |  |  |
| Proportion of Urban Children in Region 2 | 0.224 |  |  |
| Proportion of Urban Children in Region 3 | 0.213 |  |  |
| Proportion of Urban Children in Region 4 | 0.617 |  |  |
| Proportion of Urban Children in Region 5 | 0.295 |  |  |
| Proportion of Urban Children in Region 6 | 0.750 |  |  |
| Proportion of Urban Children in Lowest SES | 0.029 |  |  |
| Proportion of Urban Children in Low SES | 0.051 |  |  |
| Proportion of Urban Children in Middle SES | 0.141 |  |  |
| Proportion of Urban Children in High SES | 0.297 |  |  |
| Proportion of Urban Children in Highest SES | 0.483 |  |  |
| Proportion of Rural Children in Lowest SES | 0.299 |  |  |
| Proportion of Rural Children in Low SES | 0.323 |  |  |
| Proportion of Rural Children in Middle SES | 0.208 |  |  |
| Proportion of Rural Children in High SES | 0.129 |  |  |
| Proportion of Rural Children in Highest SES | 0.041 |  |  |
| Proportion of Lowest SES Children with Mother's with No Education | 0.872 |  |  |
| Proportion of Low SES Children with Mother's with No Education | 0.685 |  |  |
| Proportion of Middle SES Children with Mother's with No Education | 0.385 |  |  |
| Proportion of High SES Children with Mother's with No Education | 0.159 |  |  |
| Proportion of Highest SES Children with Mother's with No Education | 0.064 |  |  |
| Proportion of Lowest SES Children with Mother's with a Primary Education | 0.099 |  |  |
| Proportion of Low SES Children with Mother's with a Primary Education | 0.202 |  |  |
| Proportion of Middle SES Children with Mother's with a Primary Education | 0.289 |  |  |
| Proportion of High SES Children with Mother's with a Primary Education | 0.201 |  |  |
| Proportion of Highest SES Children with Mother's with a Primary Education | 0.084 |  |  |
| Proportion of Lowest SES Children with Mother's with a Secondary Education | 0.029 |  |  |
| Proportion of Low SES Children with Mother's with a Secondary Education | 0.114 |  |  |
| Proportion of Middle SES Children with Mother's with a Secondary Education | 0.326 |  |  |
| Proportion of High SES Children with Mother's with a Secondary Education | 0.639 |  |  |
| Proportion of Highest SES Children with Mother's with a Secondary Education | 0.852 |  |  |
| *Medication Taken by Facility* |  |  |  |
| Public Facilities |  |  |  |
| % ACTs | 44.6% |  | Nigeria MIS 2015[6] |
| % Chloroquine | 25.2% |  |  |
| % Other Treatments | 30.2% |  |  |
| Private Facilities |  |  |  |
| % ACTs | 40.9% |  |  |
| % Chloroquine | 15.7% |  |  |
| % Other Treatments | 43.4% |  |  |
| Pharmacies/Chemists |  |  |  |
| % ACTs | 34.9% |  |  |
| % Chloroquine | 28.8% |  |  |
| % Other Treatments | 36.2% |  |  |
| Drug Stores/Drug Hawkers/General Retailers |  |  |  |
| % ACTs | 50.0% |  |  |
| % Chloroquine | 8.9% |  |  |
| % Other Treatments | 41.1% |  |  |
| CHWs |  |  |  |
| % ACTs | 62.1% |  |  |
| % Chloroquine | 0.0% |  |  |
| % Other Treatments | 37.9% |  |  |
| Self-Treatment |  |  |  |
| % ACTs | 30.4% |  |  |
| % Chloroquine | 27.9% |  |  |
| % Other Treatments | 41.8% |  |  |
| *Care-Seeking* |  |  |  |
| Care-Seeking Behavior (%) |  |  |  |
| Public Facilities | 19.91% |  | Nigeria MIS 2015[6] |
| Private Facilities | 6.07% |  | Nigeria MIS 2015[6] |
| Pharmacies/Chemists | 40.06% |  |  |
| Drug Stores/Drug Hawkers/General Retailers | 0.81% |  |  |
| CHWs | 0.85% |  |  |
| Self/Neighbors | 20.19% |  |  |
| No Treatment | 12.11% |  |  |
| 20% More Seeking Care Scenario (%) |  |  |  |
| Public Facilities | 21.31% |  | Nigeria MIS 2015[6] |
| Private Facilities | 6.82% |  | Nigeria MIS 2015[6] |
| Pharmacies/Chemists | 39.65% |  |  |
| Drug Stores/Drug Hawkers/General Retailers | 1.32% |  |  |
| CHWs | 1.20% |  |  |
| Self/Neighbors | 20.79% |  |  |
| No Treatment | 8.91% |  |  |
|  |  |  |  |
| *SF and Treatment Adherence Proportions* |  |  |  |
| ACTs SF Proportions |  |  |  |
| Not SF (API > 85%) | 0.882 |  | Estimated based on: Ioset 2009[18], Onwujekwe 2009[19], Ochekpe 2010[20], Sabartova/WHO 2011[21], Kaur 2015[22], Kaur 2016[23] |
| Category 1: API = 75-85% | 0.064 |  | Estimated based on: Ioset 2009[18], Onwujekwe 2009[19], Ochekpe 2010[20], Sabartova/WHO 2011[21], Affum 2013[24], Nyarko 2014[25], ACTWatch Nigeria Outlet Survey 2015[26], Kaur 2015[22], Kaur 2016[23] |
| Category 2: API = 50-75% | 0.027 |  | Estimated based on: Ioset 2009[18], Onwujekwe 2009[19], Ochekpe 2010[20], Sabartova/WHO 2011[21], Affum 2013[24], Nyarko 2014[25], ACTWatch Nigeria Outlet Survey 2015[26], Kaur 2015[22], Kaur 2016[23] |
| Category 3: API < 50% | 0.027 |  |  |
| Chloroquine SF Proportions |  |  |  |
| Not SF (API > 85%) | 0.494 |  | Estimated based on: Taylor 2001[27], Idowu 2006[28], Aina 2008[29], Onwujekwe 2009[19] |
| Category 1: API = 75-85% | 0.273 |  | Estimated based on:  Onwujekwe 2009[19], Sabartova/WHO 2011[21], Affum 2013[24], Nyarko 2014[25], ACTWatch Nigeria Outlet Survey 2015[26], Kaur 2015[22] |
| Category 2: API = 50-75% | 0.118 |  | Estimated based on:  Onwujekwe 2009[19], Sabartova/WHO 2011[21], Affum 2013[24], Nyarko 2014[25], ACTWatch Nigeria Outlet Survey 2015[26], Kaur 2015[22] |
| Category 3: API < 50% | 0.116 |  |  |
| Other Treatments SF Proportions |  |  |  |
| Not SF (API > 85%) | 0.479 |  | Estimated based on: Taylor 2001[27], Idowu 2006[28], Aina 2008[29], Onwujekwe 2009[19], Sabartova/WHO 2011[21] |
| Category 1: API = 75-85% | 0.281 |  | Estimated based on: Onwujekwe 2009[19], Sabartova/WHO 2011[21], Affum 2013[24], Nyarko 2014[25], ACTWatch Nigeria Outlet Survey 2015[26], Kaur 2015[22], Affum 2013[24] |
| Category 2: API = 50-75% | 0.121 |  | Estimated based on: Onwujekwe 2009[19], Sabartova/WHO 2011[21], Affum 2013[24], Nyarko 2014[25], ACTWatch Nigeria Outlet Survey 2015[26], Kaur 2015[22], Affum 2013[24] |
| Category 3: API < 50% | 0.119 |  |  |
| Treatment Adherence Proportions |  |  |  |
| Good: Completes 5-6 Doses | 0.7473 |  | Bruxvoort 2015[30] |
| Fair: Completes 4 Doses | 0.1092 |  | Bruxvoort 2015[30] |
| Poor: Completes 3 Doses | 0.0733 |  |  |
| Very Poor: Completes 2 Doses | 0.0312 |  |  |
| Does Not Adhere: Completes 0-1 Dose | 0.0390 |  |  |
| *Costs* |  |  |  |
| *Patient Costs* | | | |
| Cost for Care-Seeking Base (Public/Private Facilities) | $ 1.00 | $ 1.00 | Onwujekwe 2013[31] |
| Cost for Care-Seeking Base (Pharmacies/Chemists, Drugstores/Drug Hawkers/General Retailers) | $ 0.10 | $ 0.11 | Assumed to be 10% of Public/Private Care-Seeking |
| Costs for Care for Special Foods for Child | $ 1.00 | $ 0.25 | Assumption based on: Hansen 2017[32] |
| Costs for Care for Supplemental Medicines | $ 1.00 | $ 0.25 | Assumption based on: Batwala 2011[33] |
| Average Testing Costs | $ 1.11 | $ 0.24 | Estimated based on: ACTWatch Nigeria Outlet Survey 2015[26] |
| Average Testing Costs (Public Facilities) | $ 0.00 | $ 0 | ACTWatch Nigeria Outlet Survey 2015[26] |
| Cost for Care (Public Facilities) | $ 0.30 | $ 0.24 | Nigeria MIS 2015[6] |
| Cost for Care (Private Facilities) | $ 16.28 | $ 1.25 | Jimoh 2007[34], Salawu 2016[35] |
| Additional Cost per pediatric malaria hospitalization (Private Facilities) | $ 10.24 | $ 8.20 | Onwujekwe 2013[31] |
| Productivity Loss for Seeking Care (Public/Private Facilities) | $ 9.59 | $ 7.27 | Onwujekwe 2013[31]  Assumed to be 25% of the loss of  Public/Private Facilities |
| Productivity Loss for Seeking Care (Pharmacies/Chemists, Drugstores/Drug Hawkers/General Retailers) | $ 2.40 | $ 1.82 |  |
| Productivity Loss per Sick Day | $ 6.30 |  | Estimated based on: World Bank 2016[36] |
| Productivity Losses from Death | $ 52,554.65 |  | Estimated based on: World Bank 2016[36]  Estimated based on: World Bank 2016[36], IHME 2017[37] |
| Neurological Sequelae Disability Productivity Losses | $ 21,745.56 |  |  |
| Additional Neurological Sequelae Disability Productivity Losses for a severe case | $ 6,739.06 |  | Estimated based on: World Bank 2016[36], IHME 2017[37] |
| *Patient Medication Costs* |  |  |  |
| Public Facilities |  |  |  |
| Average Cost of ACTs | $ 0.00 |  | ACTwatch Nigeria Outlet Survey 2015[26] |
| Average Cost of Chloroquine | $ 0.00 |  | ACTwatch Nigeria Outlet Survey 2015[26] |
| Average Cost of Other Treatments | $ 0.00 |  |  |
| Private Facilities |  |  |  |
| Average Cost of ACTs | $ 2.10 | ($1.53 – $2.67) |  |
| Average Cost of Chloroquine | $ 0.41 | ($0 – $0.91) |  |
| Average Cost of Other Treatments | $ 1.40 | ($0.61 – $2.19) |  |
| Pharmacies/Chemists |  |  |  |
| Average Cost of ACTs | $ 3.25 | ($2.69 – $3.81) |  |
| Average Cost of Chloroquine | $ 0.51 | ($0.01 – $1.01) |  |
| Average Cost of Other Treatments | $ 1.47 | ($0.71 – $2.23) |  |
| Drug Stores/Drug Hawkers/General Retailers |  |  |  |
| Average Cost of ACTs | $ 2.08 | ($1.66 – $2.50) |  |
| Average Cost of Chloroquine | $ 0.25 | ($0 – $0.75) |  |
| Average Cost of Other Treatments | $ 1.47 | ($0.71 – $2.23) |  |
| CHWs |  |  |  |
| Average Cost of ACTs | $ 0.00 |  |  |
| Average Cost of Chloroquine | $ 0.00 |  |  |
| Average Cost of Other Treatments | $ 0.00 |  |  |
| Self/Neighbors |  |  |  |
| Average Cost of ACTs | $ 0.00 |  | Assumption |
| Average Cost of Chloroquine | $ 0.00 |  | Assumption |
| Average Cost of Other Treatments | $ 0.00 |  |  |
| *Facility Costs* |  |  |  |
| Facility Cost per Testing | $ 1.27 |  | Estimated based on: Uzochukwu 2009[38], Onwujekwe 2013[31] |
| Facility Cost for Supplemental Medications  (Antibiotics, analgesics, etc.) | $ 3.88 |  | Ezenduka 2017[39] |
| Facility Cost per ACTs | $ 2.07 |  | Estimated based on: Onwujekwe 2013[31], Ezenduka 2017[40] |
| Facility Cost for Chloroquine | $ 0.14 |  | Management Sciences for Health [41] |
| Facility Cost per Other Treatments | $ 0.45 |  | Management Sciences for Health [41]  Estimated based on: Onwujekwe 2013[31], Ezenduka 2017[39] |
| Public Facility Cost per Case (w/o testing or medications) | $ 31.04 |  |  |
| Facility Cost per Pediatric Malaria Hospitalization (Public Facilities) | $ 92.86 |  | Estimated based on: Lubell 2011[3], Ezenduka 2017[39] |
| Cost per CHW Treatment | $ 2.90 |  | Onwujekwe 2007[42] |
| Cost per CHW Testing | $ 0.87 |  | Uzochukwu 2009[38] |
| Facility Cost per CHW visit (Program costs w/o testing and treatment) | $ 14.71 |  | Estimated based on: Onwujekwe 2007[42] |
| Opportunity Cost per CHW visit | $ 0.12 |  | Onwujekwe 2007[42] |
| *Stock-out Probabilities* |  |  |  |
| Public Facilities | 12.7% |  | ACTwatch Nigeria Outlet Survey 2015[26] |
| Private Facilities | 25.5% |  | ACTwatch Nigeria Outlet Survey 2015[26] |
| Pharmacies/Chemists | 0.1% |  |  |
| Drug Stores/Drug Hawkers/General Retailers | 11.6% |  |  |
| CHWs | 0% |  |  |

ACTs: Artemisinin-based combination therapies; API: Active pharmaceutical ingredient; CHWs: Community health workers; MIS: Malaria indicatory survey; SES: socio-economic status; SF: substandard and falsified

**References**

1. Malaria Atlas Project. Under-five malaria incidence in Nigeria 2018 [cited 2019 April 1]. Available from: https://map.ox.ac.uk/.

2. Camponovo F, Bever CA, Galactionova K, Smith T, Penny MA. Incidence and admission rates for severe malaria and their impact on mortality in Africa. Malaria journal. 2017;16(1):1. doi: 10.1186/s12936-016-1650-6.

3. Lubell Y, Staedke SG, Greenwood BM, Kamya MR, Molyneux M, Newton PN, et al. Likely health outcomes for untreated acute febrile illness in the tropics in decision and economic models; a Delphi survey. PLoS One. 2011;6(2):e17439. Epub 2011/03/11. doi: 10.1371/journal.pone.0017439. PubMed PMID: 21390277; PubMed Central PMCID: PMCPMC3044764.

4. Dondorp AM, Fanello CI, Hendriksen IC, Gomes E, Seni A, Chhaganlal KD, et al. Artesunate versus quinine in the treatment of severe falciparum malaria in African children (AQUAMAT): an open-label, randomised trial. Lancet (London, England). 2010;376(9753):1647-57. Epub 2010/11/11. doi: 10.1016/s0140-6736(10)61924-1. PubMed PMID: 21062666; PubMed Central PMCID: PMCPMC3033534.

5. Lubell Y, Dondorp A, Guerin PJ, Drake T, Meek S, Ashley E, et al. Artemisinin resistance--modelling the potential human and economic costs. Malaria journal. 2014;13:452. Epub 2014/11/25. doi: 10.1186/1475-2875-13-452. PubMed PMID: 25418416; PubMed Central PMCID: PMCPMC4254187.

6. National Malaria Elimination Programme - NMEP/Nigeria, National Population Commission - NPC/Nigeria, National Bureau of Statistics - NBS/Nigeria, ICF International. Nigeria Malaria Indicator Survey 2015. Abuja, Nigeria and Rockville MD, USA: NMEP, NPC, and ICF International, 2016.

7. Falade C, Makanga M, Premji Z, Ortmann CE, Stockmeyer M, de Palacios PI. Efficacy and safety of artemether-lumefantrine (Coartem) tablets (six-dose regimen) in African infants and children with acute, uncomplicated falciparum malaria. Transactions of the Royal Society of Tropical Medicine and Hygiene. 2005;99(6):459-67. Epub 2005/04/20. doi: 10.1016/j.trstmh.2004.09.013. PubMed PMID: 15837358.

8. Four Artemisinin-Based Combinations (4ABC) Study Group. A Head-to-Head Comparison of Four Artemisinin-Based Combinations for Treating Uncomplicated Malaria in African Children: A Randomized Trial. PLoS Medicine. 2011;8(11). doi: 10.1371/journal.pmed.1001119. PubMed PMID: 22087077; PubMed Central PMCID: PMCPMC3210754.

9. Falade C, Dada-Adegbola H, Ogunkunle O, Oguike M, Nash O, Ademowo O. Evaluation of the Comparative Efficacy and Safety of Artemether-Lumefantrine, Artesunate-Amodiaquine and Artesunate-Amodiaquine-Chlorpheniramine (Artemoclo™) for the Treatment of Acute Uncomplicated Malaria in Nigerian Children. Medical Principles and Practice. 2014;23(3):204-11. doi: 10.1159/000360578. PubMed PMID: 24732940; PubMed Central PMCID: PMCPMC5586877.

10. Grandesso F, Bachy C, Donam I, Ntambi J, Habimana J, D’Alessandro U, et al. Efficacy of chloroquine, sulfadoxine–pyrimethamine and amodiaquine for treatment of uncomplicated Plasmodium falciparum malaria among children under five in Bongor and Koumra, Chad. Transactions of the Royal Society of Tropical Medicine and Hygiene. 2006;100(5):419-26. doi: https://doi.org/10.1016/j.trstmh.2005.07.017.

11. Nahum A, Erhart A, Ahounou D, Bonou D, Van Overmeir C, Menten J, et al. Extended high efficacy of the combination sulphadoxine-pyrimethamine with artesunate in children with uncomplicated falciparum malaria on the Benin coast, West Africa. Malaria journal. 2009;8:37. Epub 2009/03/05. doi: 10.1186/1475-2875-8-37. PubMed PMID: 19257898; PubMed Central PMCID: PMCPMC2653068.

12. Adjuik M, Agnamey P, Babiker A, Borrmann S, Brasseur P, Cisse M, et al. Amodiaquine-artesunate versus amodiaquine for uncomplicated Plasmodium falciparum malaria in African children: a randomised, multicentre trial. Lancet (London, England). 2002;359(9315):1365-72. Epub 2002/04/30. PubMed PMID: 11978332.

13. Ramharter M, Oyakhirome S, Klein Klouwenberg P, Adegnika AA, Agnandji ST, Missinou MA, et al. Artesunate-clindamycin versus quinine-clindamycin in the treatment of Plasmodium falciparum malaria: a randomized controlled trial. Clinical infectious diseases : an official publication of the Infectious Diseases Society of America. 2005;40(12):1777-84. Epub 2005/05/24. doi: 10.1086/430309. PubMed PMID: 15909266.

14. Zongo I, Dorsey G, Rouamba N, Dokomajilar C, Lankoande M, Ouedraogo JB, et al. Amodiaquine, sulfadoxine-pyrimethamine, and combination therapy for uncomplicated falciparum malaria: a randomized controlled trial from Burkina Faso. The American journal of tropical medicine and hygiene. 2005;73(5):826-32. Epub 2005/11/12. PubMed PMID: 16282288.

15. Faucher JF, Aubouy A, Adeothy A, Cottrell G, Doritchamou J, Gourmel B, et al. Comparison of sulfadoxine-pyrimethamine, unsupervised artemether-lumefantrine, and unsupervised artesunate-amodiaquine fixed-dose formulation for uncomplicated plasmodium falciparum malaria in Benin: a randomized effectiveness noninferiority trial. The Journal of infectious diseases. 2009;200(1):57-65. Epub 2009/05/28. doi: 10.1086/599378. PubMed PMID: 19469703.

16. Verret WJ, Arinaitwe E, Wanzira H, Bigira V, Kakuru A, Kamya M, et al. Effect of Nutritional Status on Response to Treatment with Artemisinin-Based Combination Therapy in Young Ugandan Children with Malaria. Antimicrobial Agents and Chemotherapy. 2011;55(6):2629-35. doi: 10.1128/aac.01727-10.

17. Yeka A, Tibenderana J, Achan J, D'Alessandro U, Talisuna AO. Efficacy of quinine, artemether-lumefantrine and dihydroartemisinin-piperaquine as rescue treatment for uncomplicated malaria in Ugandan children. PLoS One. 2013;8(1):e53772. Epub 2013/01/26. doi: 10.1371/journal.pone.0053772. PubMed PMID: 23349741; PubMed Central PMCID: PMCPMC3551967.

18. Ioset JR, Kaur H. Simple field assays to check quality of current artemisinin-based antimalarial combination formulations. PLoS One. 2009;4(9):e7270. Epub 2009/10/01. doi: 10.1371/journal.pone.0007270. PubMed PMID: 19789707; PubMed Central PMCID: PMCPMC2749338.

19. Onwujekwe O, Kaur H, Dike N, Shu E, Uzochukwu B, Hanson K, et al. Quality of anti-malarial drugs provided by public and private healthcare providers in south-east Nigeria. Malaria journal. 2009;8:22. Epub 2009/02/12. doi: 10.1186/1475-2875-8-22. PubMed PMID: 19208221; PubMed Central PMCID: PMCPMC2649149.

20. Ochekpe NA, Agbowuro AA, Attah SE. Correlation of price and quality of medicines: Assessment of some artemisinin antimalarials in nigeria based on gphf minilab. International Journal of Drug Development and Research. 2010;2(1):211-8.

21. Sabartova JT, Amor Survey of the quality of selected antimalarial medicines circulating in six countries of sub-Saharan Africa Geneva: 2011.

22. Kaur H, Allan EL, Mamadu I, Hall Z, Ibe O, Sherbiny ME, et al. Quality of artemisinin-based combination formulations for malaria treatment: Prevalence and risk factors for poor quality medicines in public facilities and private sector drug outlets in Enugu, Nigeria. PLoS ONE. 2015;10(5). doi: 10.1371/journal.pone.0125577.

23. Kaur H, Clarke S, Lalani M, Phanouvong S, Guerin P, McLoughlin A, et al. Fake anti-malarials: start with the facts. Malaria journal. 2016;15:86. Epub 2016/02/14. doi: 10.1186/s12936-016-1096-x. PubMed PMID: 26873700; PubMed Central PMCID: PMCPMC4752758.

24. Affum AO, Lowor S, Osae SD, Dickson A, Gyan BA, Tulasi D. A pilot study on quality of artesunate and amodiaquine tablets used in the fishing community of Tema, Ghana. Malaria journal. 2013;12:220. Epub 2013/07/03. doi: 10.1186/1475-2875-12-220. PubMed PMID: 23809666; PubMed Central PMCID: PMCPMC3722045.

25. Nyarko SH, Cobblah A. Sociodemographic Determinants of Malaria among Under-Five Children in Ghana. Malaria research and treatment. 2014;2014:304361. Epub 2015/01/13. doi: 10.1155/2014/304361. PubMed PMID: 25580349; PubMed Central PMCID: PMCPMC4279724.

26. ACTwatch Group. ACTwatch Study Reference Document: The Federal Republic of Nigeria Outlet Survey 2015. Washington, DC: PSI, 2015.

27. Taylor RB, Shakoor O, Behrens RH, Everard M, Low AS, Wangboonskul J, et al. Pharmacopoeial quality of drugs supplied by Nigerian pharmacies. Lancet (London, England). 2001;357(9272):1933-6. Epub 2001/06/27. PubMed PMID: 11425415.

28. Idowu OA, Apalara SB, Lasisi AA. Assessment of quality of chloroquine tablets sold by drug vendors in Abeokuta, Nigeria. Tanzania health research bulletin. 2006;8(1):45-6. Epub 2006/10/25. PubMed PMID: 17058801.

29. Aina BA, Tayo F, Taylor O. Cost implication of irrational prescribing of chloroquine in Lagos State general hospitals. Journal of infection in developing countries. 2008;2(1):68-72. Epub 2008/01/01. PubMed PMID: 19736391.

30. Bruxvoort K, Kalolella A, Cairns M, Festo C, Kenani M, Lyaruu P, et al. Are Tanzanian patients attending public facilities or private retailers more likely to adhere to artemisinin-based combination therapy? Malaria journal. 2015;14:87. Epub 2015/04/19. doi: 10.1186/s12936-015-0602-x. PubMed PMID: 25889767; PubMed Central PMCID: PMCPMC4340668.

31. Onwujekwe O, Uguru N, Etiaba E, Chikezie I, Uzochukwu B, Adjagba A. The economic burden of malaria on households and the health system in Enugu State southeast Nigeria. PLoS One. 2013;8(11):e78362. Epub 2013/11/14. doi: 10.1371/journal.pone.0078362. PubMed PMID: 24223796; PubMed Central PMCID: PMCPMC3817251.

32. Hansen KS, Clarke SE, Lal S, Magnussen P, Mbonye AK. Cost-effectiveness analysis of introducing malaria diagnostic testing in drug shops: A cluster-randomised trial in Uganda. PLOS ONE. 2017;12(12):e0189758. doi: 10.1371/journal.pone.0189758.

33. Batwala V, Magnussen P, Hansen KS, Nuwaha F. Cost-effectiveness of malaria microscopy and rapid diagnostic tests versus presumptive diagnosis: implications for malaria control in Uganda. Malaria journal. 2011;10:372. Epub 2011/12/21. doi: 10.1186/1475-2875-10-372. PubMed PMID: 22182735; PubMed Central PMCID: PMCPMC3266346.

34. Jimoh A, Sofola O, Petu A, Okorosobo T. Quantifying the economic burden of malaria in Nigeria using the willingness to pay approach. Cost effectiveness and resource allocation : C/E. 2007;5:6-. doi: 10.1186/1478-7547-5-6. PubMed PMID: 17517146.

35. Salawu AT, Fawole OI, Dairo MD. PATRONAGE AND COST OF MALARIA TREATMENT IN PRIVATE HOSPITALS IN IBADAN NORTH L.G.A SOUTH WESTERN, NIGERIA. Annals of Ibadan postgraduate medicine. 2016;14(2):81-4. Epub 2016/01/01. PubMed PMID: 28337092; PubMed Central PMCID: PMCPMC5354625.

36. The World Bank. Nigeria: Data 2016 [cited 2019 April 1]. Available from: https://data.worldbank.org/country/nigeria.

37. Global Burden of Disease [Internet]. 2017. Available from: <http://ghdx.healthdata.org/record/global-burden-disease-study-2017-gbd-2017-disability-weights>.

38. Uzochukwu BS, Obikeze EN, Onwujekwe OE, Onoka CA, Griffiths UK. Cost-effectiveness analysis of rapid diagnostic test, microscopy and syndromic approach in the diagnosis of malaria in Nigeria: implications for scaling-up deployment of ACT. Malaria journal. 2009;8:265. doi: 10.1186/1475-2875-8-265. PubMed PMID: 19930666; PubMed Central PMCID: PMC2787522.

39. Ezenduka CC, Falleiros DR, Godman BB. Evaluating the Treatment Costs for Uncomplicated Malaria at a Public Healthcare Facility in Nigeria and the Implications. PharmacoEconomics - open. 2017;1(3):185-94. doi: 10.1007/s41669-017-0021-8. PubMed PMID: 29441495; PubMed Central PMCID: PMC5691839.

40. Ezenduka CC, Falleiros DR, Godman BB. Evaluating the Treatment Costs for Uncomplicated Malaria at a Public Healthcare Facility in Nigeria and the Implications. PharmacoEconomics - open. 2017;1(3):185-94. doi: 10.1007/s41669-017-0021-8. PubMed PMID: 29441495.

41. International Medical Products Price Guide [Internet]. [cited 2018]. Available from: <http://mshpriceguide.org/en/home/>.

42. Onwujekwe O, Uzochukwu B, Ojukwu J, Dike N, Shu E. Feasibility of a community health worker strategy for providing near and appropriate treatment of malaria in southeast Nigeria: an analysis of activities, costs and outcomes. Acta tropica. 2007;101(2):95-105. doi: 10.1016/j.actatropica.2006.07.013. PubMed PMID: 17270139.
