# Supplementary figures and images for: The economic impact of substandard and falsified antimalarial medications in Nigeria
Source: PLoS One. 2019 Aug 15;14(8):e0217910. doi: 10.1371/journal.pone.0217910 (PMC6695148; doi:10.1371/journal.pone.0217910)

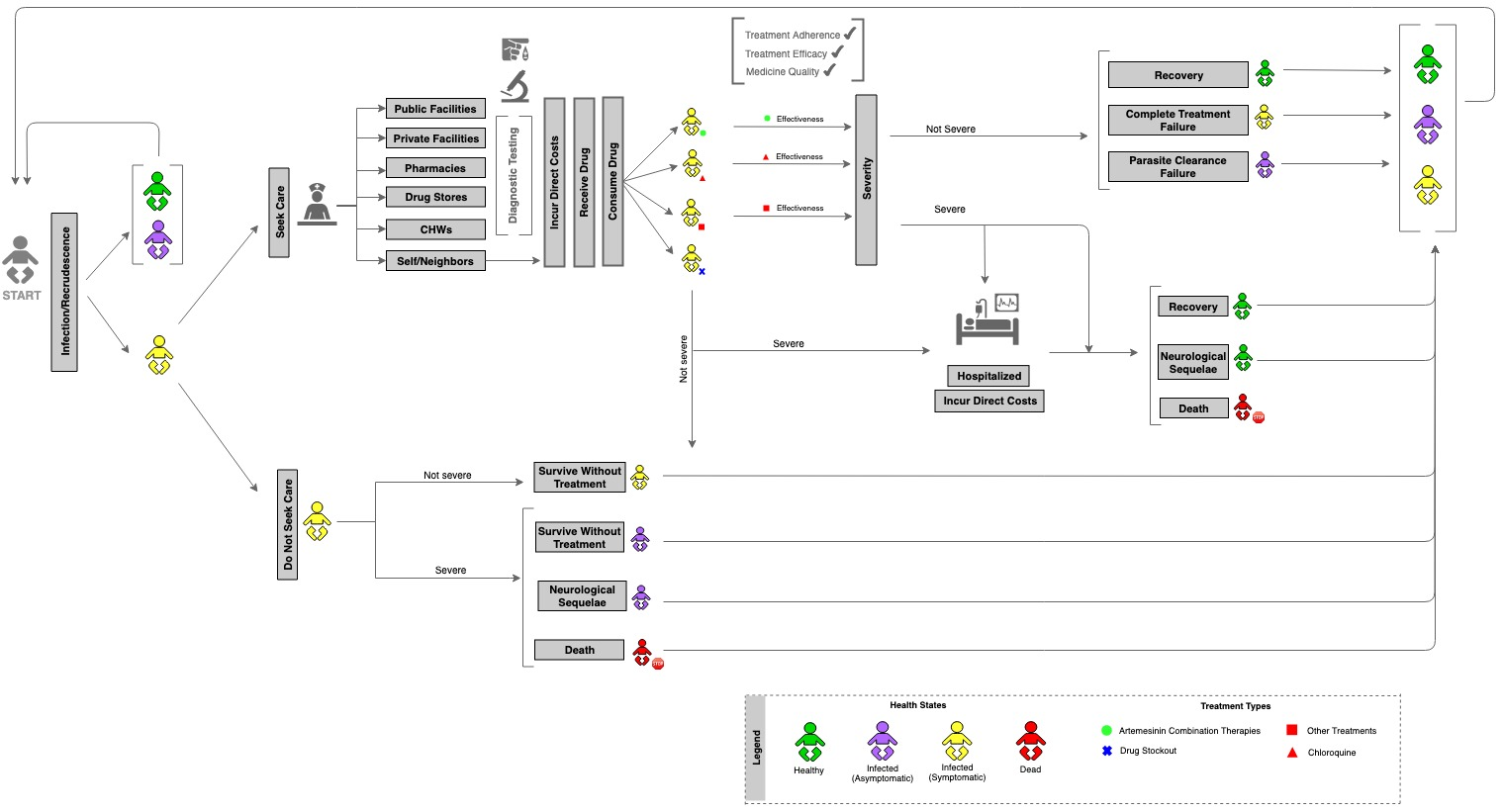

Supplement: S1 Fig — (TIF) [file pone.0217910.s002.tif]
